# Transcriptomic analysis of the red seaweed *Laurencia dendroidea* (Florideophyceae, Rhodophyta) and its microbiome

**DOI:** 10.1186/1471-2164-13-487

**Published:** 2012-09-17

**Authors:** Louisi Souza de Oliveira, Gustavo Bueno Gregoracci, Genivaldo Gueiros Zacarias Silva, Leonardo Tavares Salgado, Gilberto Amado Filho, Marcio Alves-Ferreira, Renato Crespo Pereira, Fabiano L Thompson

**Affiliations:** 1 Departamento de Biologia Marinha, Instituto de Biologia, Universidade Federal do Rio de Janeiro (UFRJ) Av. Carlos Chagas Filho, 373-CCS - IB - BLOCO A (ANEXO) A3- 202, Rio de Janeiro, 21941-599, Brazil; 2 Laboratório de Bioinformática e Biologia Evolutiva, Universidade Federal de Pernambuco. Av. Prof. Moraes Rego 1235, Cidade Universitária, Recife, 50670-901, PE, Brazil; 3 Instituto de Pesquisa Jardim Botânico do Rio de Janeiro, Rua Pacheco Leão, 915. Jardim Botânico, Rio de Janeiro, 22460-030, RJ, Brazil; 4 Departamento de Genética. Instituto de Biologia. Av. Prof. Rodolpho Paulo Rocco, s/n, CCS, Sala A2-93, Universidade Federal do Rio de Janeiro (UFRJ), Rio de Janeiro, 21941-599, RJ, Brazil; 5 Departamento de Biologia Marinha, Universidade Federal Fluminense (UFF). Morro do Valonguinho, s/n. Centro, Niteroi, 24001-970, RJ, Brazil

**Keywords:** Red seaweed, Terpene, Bacteria, Holobiont, Metabolic pathway, EST

## Abstract

**Background:**

Seaweeds of the *Laurencia* genus have a broad geographic distribution and are largely recognized as important sources of secondary metabolites, mainly halogenated compounds exhibiting diverse potential pharmacological activities and relevant ecological role as anti-epibiosis. Host-microbe interaction is a driving force for co-evolution in the marine environment, but molecular studies of seaweed-associated microbial communities are still rare. Despite the large amount of research describing the chemical compositions of *Laurencia* species, the genetic knowledge regarding this genus is currently restricted to taxonomic markers and general genome features. In this work we analyze the transcriptomic profile of *L*. *dendroidea* J. Agardh, unveil the genes involved on the biosynthesis of terpenoid compounds in this seaweed and explore the interactions between this host and its associated microbiome.

**Results:**

A total of 6 transcriptomes were obtained from specimens of *L. dendroidea* sampled in three different coastal locations of the Rio de Janeiro state. Functional annotations revealed predominantly basic cellular metabolic pathways. Bacteria was the dominant active group in the microbiome of *L*. *dendroidea*, standing out nitrogen fixing Cyanobacteria and aerobic heterotrophic Proteobacteria. The analysis of the relative contribution of each domain highlighted bacterial features related to glycolysis, lipid and polysaccharide breakdown, and also recognition of seaweed surface and establishment of biofilm. Eukaryotic transcripts, on the other hand, were associated with photosynthesis, synthesis of carbohydrate reserves, and defense mechanisms, including the biosynthesis of terpenoids through the mevalonate-independent pathway.

**Conclusions:**

This work describes the first transcriptomic profile of the red seaweed *L*. *dendroidea*, increasing the knowledge about ESTs from the Florideophyceae algal class. Our data suggest an important role for *L. dendroidea* in the primary production of the holobiont and the role of Bacteria as consumers of organic matter and possibly also as nitrogen source. Furthermore, this seaweed expressed sequences related to terpene biosynthesis, including the complete mevalonate-independent pathway, which offers new possibilities for biotechnological applications using secondary metabolites from *L. dendroidea*.

## Background

*Laurencia dendroidea* is a red seaweed species widespread in the Atlantic Ocean, whose type locality is in Brazil. It is found from the intertidal to the subtidal zone at 3m depth. The thalli are erect, forming dense tufts 4–20 cm high, brown-purple or violet-greenish in color [1]. The genus *Laurencia*[2] was recognized, since the first studies on natural products in the 1960s, as an important source of secondary metabolites, mainly halogenated compounds [3,4]. The secondary metabolites of *Laurencia* play a relevant ecological role as chemical defenses against bacterial colonization and infection [5-7].

Seaweeds are especially susceptible to microbial colonization due to the biosynthesis and release of large amounts of organic compounds, which may serve as chemo-attractants and nutrient source for microbes [8]. In this context, secondary metabolites and exudates may act together selecting the microbial community associated with the surfaces and tissues of seaweeds [9,10]. Host-microbe interaction is widely recognized as one of the main driving forces for co-evolution in the marine environment, leading to the establishment of beneficial microbiomes. For instance, microbes associated with seaweed tissues may possess the ability to fix nitrogen, mineralize the organic substrates and also supply the seaweeds with carbon dioxide and growth factors [11-14]. The microbiome on seaweeds tends to be species-specific and different from the surrounding seawater [15]. However, the characterization of the microbial community living at the surface of macroalgae is still limited and the molecular studies of these communities are rare [15-17].

The untapped diversity of the secondary metabolites of *Laurencia*, particularly terpenes, has attracted considerable attention of different research groups worldwide. The pharmacological potential of these compounds comprises the strong antibiotic [18,19], antiviral [20], antimalarial [21], antitrypanosomal [22], antileishmanial [23], anti-inflammatory [24] and anti-carcinoma [25-27] activities. A major secondary metabolite of *L*. *dendroidea* is the sesquiterpene (C15) (-)-elatol, a substance that has a high biocidal and anti-epibiosis activity and could be used for the preparation of antifouling paints, or for the development of antimicrobials [28-30]. A first attempt for the commercial application of (-)-elatol resulted in the filing of the patent in Brazil to use this compound as an antifouling agent. However, technological developments are still needed to ensure its commercial viability [31]. This obstacle stems from the low yield of the extraction process, the complexity of the organic total synthesis of (-)-elatol in laboratory [32], and the failure of the large-scale cultivation of this species. A possible alternative to circumvent this problem is the synthesis of (-)-elatol in the laboratory using genetically modified organisms [31]. The cellular location and the environmental factors that induce the production of this compound by *L*. *dendroidea* are known [33,34], but the genes involved in the biosynthesis of this compound were not yet determined, representing a new research frontier in the technological use of (-)-elatol. Recent studies have determined some of the genes responsible for the biosynthesis of terpenes (i.e. cyclases or synthases) in bacteria [35], fungi [36], and plants [37]. The sequence homology observed among at least some classes of terpene synthases from these organisms [38] may facilitate the search for homolog genes in *L*. *dendroidea*.

Despite the large number of studies based on the chemical composition of *Laurencia* species, the genetic knowledge regarding this genus is currently restricted to taxonomic markers [39,40]. The genome size of *L. dendroidea* is estimated to be about 833 Mbp, based on a study of another species of the same genus [41], but gene sequences from this species have not previously been described. In this work we analyze the transcriptomic profile of *L*. *dendroidea* at different geographic locations, unveil the genes involved on the biosynthesis of terpenoid compounds in this seaweed and also explore the interactions between the alga and the associated microbiome.

## Methods

### Specimens collection

**Figure 1:**
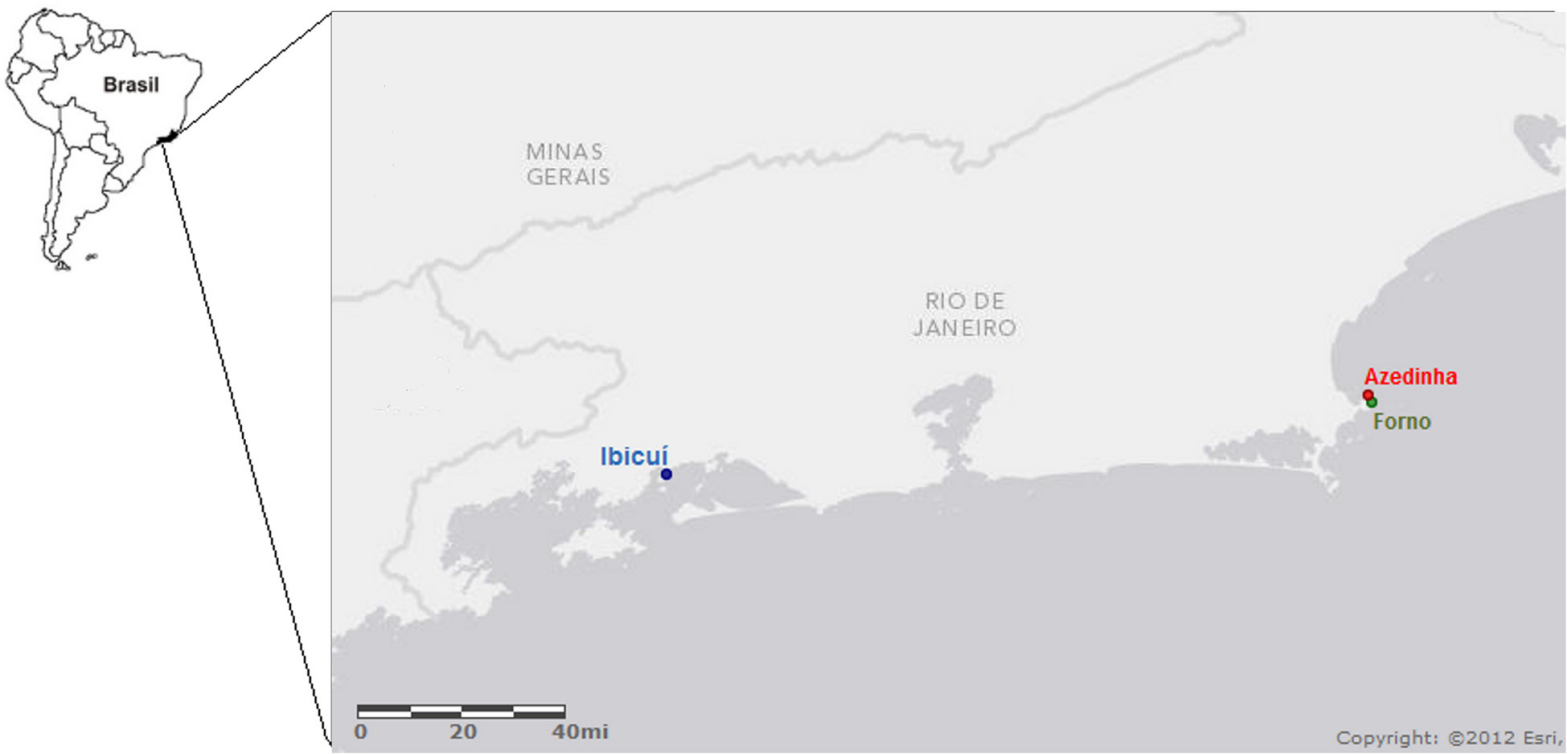
**Collection sites of specimens of *L*. *dendroidea *in Búzios and Mangaratiba, on the coast of the Rio de Janeiro state, Brazil.** Scale bar presented in miles (mi)

Specimens of *L*. *dendroidea* were randomly collected in the intertidal zone during high tide at Azedinha (22°44’28.76”S, 41°52’55.70”W) and Forno beaches (22°45’42.72”S, 41°52’29.81”W), both in Búzios, and at Ibicuí beach (22°57’45.02”S, 41°01’29.05”W) located in Mangaratiba, all these places on the coast of the Rio de Janeiro state, Brazil (Figure 1). Seaweeds were collected from nearly the same depth in two subsequent days, at approximately the same hour, with the same climatic characteristics to minimize the variation in abiotic factors. The collected thalli were rapidly cleaned of macroscopic epiphytes using tweezers, without damage to the host seaweed, and the samples were immediately frozen in liquid nitrogen, to better preserve the holobiont.

### RNA extraction, reverse-transcription and pyrosequencing

Two specimens of. *L. dendroidea* from each location were separately ground in liquid nitrogen using a mortar and pestle to obtain a fine powder. Then, 100 mg of powder from each sample was suspended in 1 mL of extraction buffer (6.5 M guanidinium hydrochloride, 100 mM Tris-HCl pH 8.0, 0.1 M sodium acetate pH 5.5, 0.1 M β-mercaptoethanol, 0.2 M KOAc). Total RNA was extracted following the method previously proposed for another red seaweed [42], but we performed an extra centrifugation step and transferred the supernatant phase before adding the chloroform, which improved the RNA quality. In order to eliminate DNA residues, all the samples were treated with DNAse (RNAse free, PROMEGA, Madison, USA). The double-stranded cDNAs (ds cDNAs) were synthesized and amplified using the SMARTer cDNA synthesis kit and the Advantage2 polymerase (Clontech, Califórnia, USA) starting from 1 μg of total RNA. The optimal number of amplification cycles was determined to be 23. This amplification did not exclude the prokaryotic portion of the holobiont, allowing the study of the microbiome along with the host. The PCR amplification products were purified using the NucleoSpin® Extract II kit (Macherey-Nagel, Düren, Alemanha). Finally the ds cDNAs were eluted in TE buffer (10 mM Tris-HCl pH 7.6; 1 mM EDTA) and sequenced using 454 pyrosequencing technology [43].

### Transcriptome analysis

The sequences from each sample were preprocessed using the software Prinseq [44] to trim poly-A/T tails at least 20 bp long and to remove reads shorter than 75 bp, and then assembled into contigs using the Roche's algorithm Newbler (minimum overlap length = 40 bp, minimum overlap identity = 95%). In our analysis we annotated both contigs and singlets after assembly (hereafter referred as transcripts), since they contained different sequences and relevant information. We downloaded all the EST sequences deposited for the class Florideophyceae in the NCBI (comprising 11 species) and assembled the reads using the TGICL software from TIGR [45]. Afterwards, the assembly of all sequences derived from *L*. *dendroidea* was aligned against the Florideophyceae EST NCBI database using the Promer alignment tool (MUMmer 3.0) using the ‘maxmatch’ parameter [46]. The results were parsed using the show-coords script with - k and - r parameters and only reciprocal matches were considered for calculations. Sequences annotated as Bacteria were treated separately in this analysis, but eventual micro-eukaryotic sequences could not be removed, since the database is not complete regarding eukaryotic marine life and no *Laurencia* sequences aside from taxonomic markers are available.

Taxonomic and functional analysis were performed on assembled sequences from all samples, using the Newbler software, and automatically annotated, using the MG-RAST server, through BLAST, against the GenBank, COG, KEGG and Subsystems databases with maximum e-value cutoff of 10^-5^[47]. The sequences obtained in this project are publicly available in the MG-RAST database and were organized in a file for each sample, named according to the site of origin, and a file containing the assembler of all reads (http://metagenomics.anl.gov/linkin.cgi?project=1274). To characterize the major phenotypic features of the microbial community associated with *L. dendroidea*, features of bacterial genera identified against Genbank (through MG-Rast) were manually annotated using the Bergey’s manuals of Systematic Bacteriology (2^nd^ ed.). Additionally, we explored the relative contributions of Bacteria and Eukarya to the functional profile. Sequences annotated against the Genbank corresponding to these domains were extracted using the Workbench tool from MG-RAST server, and re-annotated against functional hierarchies (COG, Subsystems). The functional profiles of the domains were compared using the Statistical Analysis of Metagenomic Profiles (STAMP) bioinformatics software v2.0 [48]. Statistical significance (p < 0.05) was calculated pairwise using two-sided G-test (with Yates’ correction) and Fisher’s exact test, and the confidence intervals for each proportion were calculated using the asymptotic method with a continuity correction considering the threshold of 95%. Furthermore, a specific search for two profiles using hidden markov models was performed, through the HMMER 3.0 software [49]. The first HMM profile was based on the alignment of all vanadium bromoperoxidases deposited in the protein database of NCBI, and the second one, based on the universal metal-binding domain of terpene synthases (PF03936), was obtained from PFAM as previously described [35].

## Results

**Figure 2:**
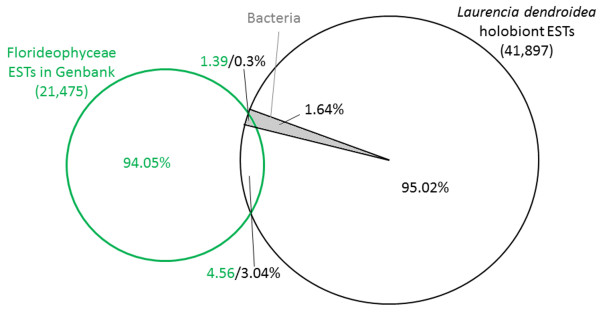
**MUMMER-based identification of shared sequences between this study and the dbEST for the class Florideophyceae.** The shaded area corresponds to sequences annotated as bacteria in this study

A total of 6 transcriptomes (235,572 reads, 52 Mbp) were obtained for specimens of the seaweed *L*. *dendroidea* originated from three different locations in the Rio de Janeiro coast. The assembly of the sequences from each replicate resulted on 500–1,000 contigs and 10,000–16,000 singlets (see Table 1 for detailed information). The COG functional annotation and the GenBank taxonomic annotation indicated that the transcriptomic profile of *L*. *dendroidea* was highly similar among the samples (Additional files 1 and 2). Since no significant differences were observed, all the reads of the 6 transcriptomes were assembled in order to represent a transcriptomic profile for this species, resulting on 3,887 contigs and 38,010 singlets. A total of 30,585 tentative unigenes (73% of the transcripts) were identified as genes coding for proteins with unknown function, indicating the need for further molecular studies in order to unravel the function of a large portion of the transcriptome of this seaweed. The closest red algal genus with sequences deposited in the database is *Griffithsia*, classified in the order Ceramiales, for which we found only 1,277 ESTs, most of them (99.76%) derived from *Griffithsia okiensis*[50]. Searching at a higher taxonomic level, the total number of ESTs from the class Florideophyceae deposited in NCBI was 37,198, comprising 21,475 unigenes, from which only 5.95% matched with 3.34% unigenes from this study (Figure 2). These numbers include the sequences of Bacteria associated with the *Laurencia* holobiont (1.94%), from which 0.3% matched with 1.39% of the sequences in the Florideophyceae database, indicating that the reference database itself contains bacterial sequences. Excluding those bacterial sequences from our analysis, 3.04% of the remaining sequences are left matching 4.56% of sequences from the Florideophyceae database (Figure 2). Therefore, 95.02% of the sequences provided by this work could potentially enrich our current knowledge regarding Florideophyceae as they represent unknown genes.

**Table 1:** **Characteristics of the sequencing and assembly of the cDNA libraries from the *Laurencia dendroidea *holobiont**

**Location**	**Azedinha1**	**Azedinha2**	**Forno1**	**Forno2**	**Ibicuí1**	**Ibicuí2**
Total Nucleotides (basepairs)	11,635,249	9,384,269	11,049,671	7,101,334	5,550,607	8,011,563
N. of Sequences	51,592	42,577	49,001	31,434	24,423	36,545
N. of Contigs	1,079	926	985	556	586	683
Avg. Size of Contigs	492.24 ± 190.19	489.62 ± 195.59	481.88 ± 195.80	466.06 ± 182.92	465.71 ± 164.32	487.58 ± 193.41
N. of Singlets	15,755	14,480	14,830	10,935	10,522	11,719
Avg. Size of Singlets	202.17 ± 78.19	198.52 ± 74.80	198.01 ± 77.76	197.09 ± 76.42	212.72 ± 80.09	195.50 ± 75.30

### Major groups of transcripts of *L*. *dendroidea*

**Figure 3:**
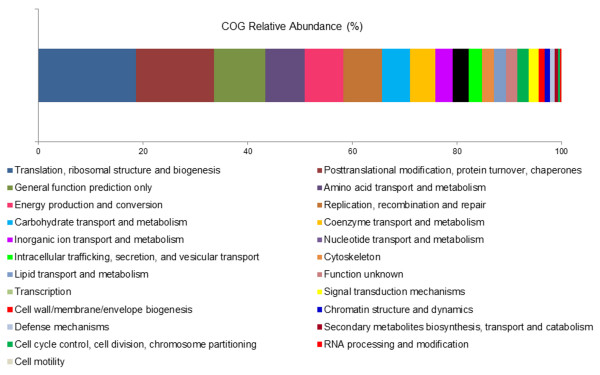
**COG functional profile overview of the transcriptome of *L*. *dendroidea***

**Figure 4:**
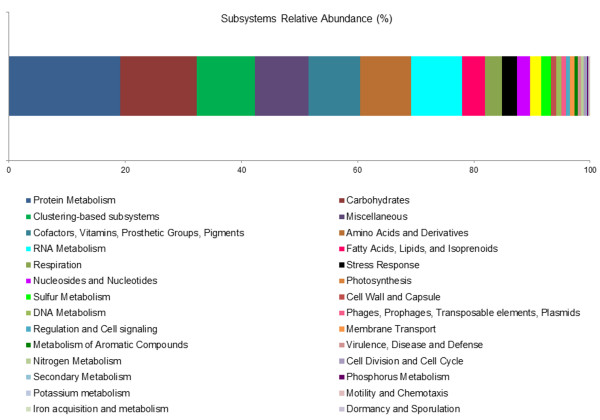
**Subsystems functional profile overview of the transcriptome of *L*. *dendroidea***

The functional classification of the ESTs revealed that most of the transcripts were related to the basal metabolism of the *Laurencia* holobiont (Figures 3 and 4). The most represented COG categories were associated to Translation, Ribosomal Structure and Biogenesis (18.65%), Posttranslational Modification, Protein Turnover and Chaperones (14.90%), and Amino acid Transport and Metabolism (7.57%). Additionally, functions associated with Energy Production and Conversion were relatively common (7.37%). Moreover, the sequences related to Replication, Recombination and Repair (7.37%), and the ESTs involved in Carbohydrate Transport and Metabolism (5.42%) were among the most represented categories in the transcriptome of *L*. *dendroidea* (Figure 3). The Subsystems annotation corroborated further the general expression profile of *Laurencia*. The main recognized features are Protein Metabolism (19.20%) and Carbohydrates (13.11%). Transcripts related to Cofactors, Vitamins, Prosthetic Groups, Pigments (8.88%), Amino Acids and Derivatives (8.77%) and RNA Metabolism (8.71%) were also numerous (Figure 4).

### Transcriptome of *L*. *dendroidea*-associated microbiome

The functional analysis of the transcriptome revealed bacterial genes that are important for surface colonization, such as the transcripts related to flagellum (0.11% of the total), CheY-like receiver domain (0.04% of the total), and S-adenosylmethionine synthetase (0.03% of the total). Indeed, we detected fewer sequences involved in Motility and Chemotaxis (0.11% of the total) in comparison with the ones related to Capsular and extracellular polysaccharides (0.53% of the total).

**Figure 5:**
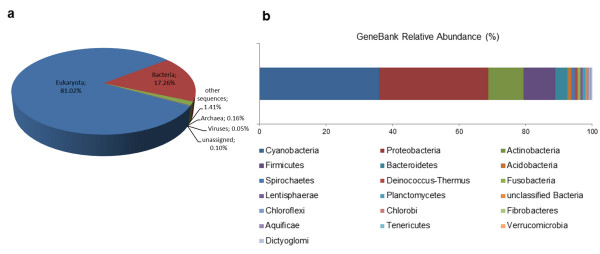
**Taxonomic classification for the transcriptome of *L*. *dendroidea. *(a)** Taxonomy overview. **(b)** Relative abundance of bacterial phyla

A total of 6,154 reads (14.69% of the total) were assigned to taxonomic categories using the GenBank database. Among them, 17.26% were classified in the domain Bacteria (Figure 5a). The most abundant bacterial transcripts were assigned to the phylum Cyanobacteria (35.97%), mainly to the orders Chroococcales, Oscillatoriales and Nostocales. The second most represented phylum is Proteobacteria (32.86%) with Gammaproteobacteria and Alphaproteobacteria as the dominant classes (Figure 5b).

Manual annotation revealed the majority of the bacterial transcripts (to which a description of respiratory metabolism could be found in Bergey’s manuals) as ascribed to aerobic (62.30%) or aerotolerant groups (14.00%). We also verified a higher abundance of transcripts related to respiration (2.96%) in comparison with the ones involved in the fermentative metabolism (0.64%). Furthermore, Bacteria expressed genes, such as Superoxide dismutase (0.51%), Glutaredoxins (0.42%), Alkyl hydroperoxide reductase (0.21%), and the chaperones GroEL (3.17%), DnaJ (1.37%) and DnaK (0.84%), related to protection from reactive oxygen species produced during aerobic metabolism (Additional file 3).

Genes involved in Photosynthesis (3.18%) and in the biosynthesis of starch (0.66%) were more abundant in eukaryotes, while ESTs related to Carbohydrate (5.63%) and Lipid Transport and Metabolism (3.58%), and to Energy Production and Conversion (11.38%) were more represented in Bacteria. Transcripts associated to Amino acid metabolism (11.50%) were also more represented in Bacteria, except for the glutamate biosynthesis that was preferentially expressed by Eukarya (0.58%, Additional file 3).

Additionally, several transcripts were attributed to bacterial genera known to be heterotrophs ( > 51.9%) or motile ( > 28.4%). Furthermore, 25.4% of the heterotroph-associated transcripts belong to genera recognized as pathogens or closely associated to eukaryotes. Along this context, the Hmmer search for vanadium-dependent bromoperoxidases, which could be involved in response to infection, resulted on 10 hits, and their functional classification was confirmed by Blastx.

### Terpenoid biosynthesis in the holobiont

**Figure 6:**
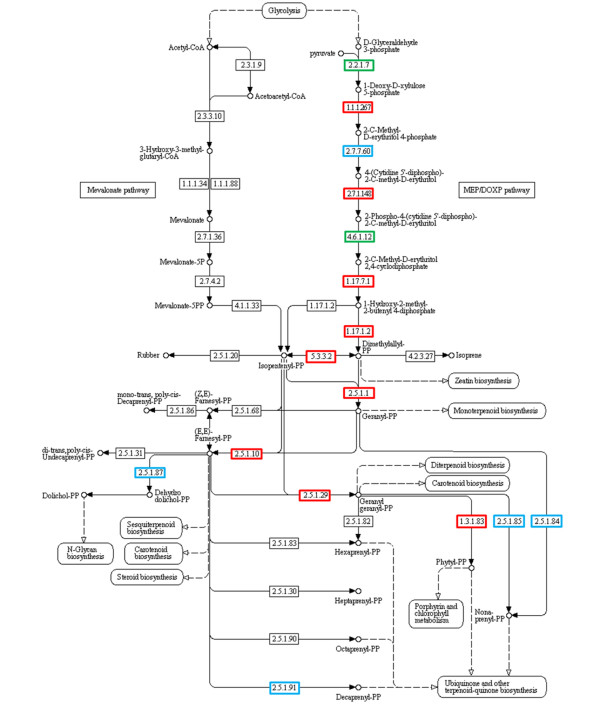
**Terpenoid backbone biosynthetic pathway.** Blue squares represent the genes identified through the KEGG database, green squares points the genes identified using the SEED database and red squares highlight the genes identified using both databases

Within the functional annotations, 34 transcripts associated to the terpenoid backbone biosynthesis in *L*. *dendroidea* were found, representing all the required enzymes involved in the mevalonate-independent pathway (Table 2, Figure 6). The identified genes participate in important steps for the synthesis of dimethylallyl diphosphate (EC 2.2.1.7; EC: 1.1.1.267; EC: 2.7.7.60; EC: 2.7.1.148; EC 4.6.1.12; EC: 1.17.7.1; EC: 1.17.1.2), its isomerization to isopentenyl diphosphate (EC: 5.3.3.2), and the condensation of these two C5-units, through the action of prenyltransferases, generating geranyl diphosphate (GDP, EC: 2.5.1.1), farnesyl diphosphate (FDP, EC: 2.5.1.10), and geranylgeranyl diphosphate (GGDP, EC: 2.5.1.29). We also found genes involved in the subsequent steps to the synthesis of chlorophylls (EC: 1.3.1.83), plastoquinone, phylloquinone, ubiquinone (EC: 2.5.1.84, EC: 2.5.1.85, EC: 2.5.1.91) and N-glycans, (EC: 2.5.1.87). The Hmmer search for the metal binding conserved domain (PF03936) in the transcriptome of *L*. *dendroidea* resulted on 3 hits, and the subsequent manual annotation confirmed their classification as terpene synthases.

**Table 2:** Description of the enzymes involved on terpenoid backbone biosynthesis

**Enzyme codes**	**Enzyme names**	**Databases**
EC 2.2.1.7	1-deoxy-D-xylulose-5-phosphate synthase.	SEED
EC: 1.1.1.267	1-deoxy-D-xylulose-5-phosphate reductoisomerase.	KEGG/SEED
EC: 2.7.7.60	2-C-methyl-D-erythritol 4-phosphate cytidylyltransferase	KEGG
EC: 2.7.1.148	4-(cytidine 5'-diphospho)-2-C-methyl-D-erythritol kinase.	KEGG/SEED
EC 4.6.1.12	2-C-methyl-D-erythritol 2,4-cyclodiphosphate synthase	SEED
EC: 1.17.7.1	(E)-4-hydroxy-3-methylbut-2-enyl-diphosphate synthase	KEGG/SEED
EC: 1.17.1.2	4-hydroxy-3-methylbut-2-enyl diphosphate reductase	KEGG/SEED
EC: 5.3.3.2	Isopentenyl-diphosphate Delta-isomerase.	KEGG/SEED
EC: 2.5.1.1	Dimethylallyltranstransferase.	KEGG/SEED
EC: 2.5.1.10	(2E,6E)-farnesyl diphosphate synthase.	KEGG/SEED
EC: 2.5.1.29	Geranylgeranyl diphosphate synthase	KEGG/SEED
EC: 2.5.1.87	Ditrans,polycis-polyprenyl diphosphate synthase ((2E,6E)-farnesyl diphosphate specific)	KEGG
EC: 1.3.1.83	Geranylgeranyl diphosphate reductase.	KEGG/SEED
EC: 2.5.1.85	All-trans-nonaprenyl-diphosphate synthase (geranylgeranyl-diphosphate specific)	KEGG
EC: 2.5.1.84	All-trans-nonaprenyl-diphosphate synthase (geranyl-diphosphate specific)	KEGG
EC: 2.5.1.91	All-trans-decaprenyl-diphosphate synthase.	KEGG

## Discussion

The present study provides the largest transcriptome dataset for the class Florideophyceae and represents the first transcriptomic characterization of the seaweed *Laurencia dendroidea*. The presented numbers could be an overestimate of the contribution of *L*. *dendroidea* to the Florideophyceae database, since we worked with complex samples. Nevertheless, at least some of the sequencing projects in the Florideophyceae dbEST are also based on non-axenic field samples [51,52], hampering the achievement of a more accurate estimate. Indeed, it is notable the presence of sequences deposited in this database that matched our bacterial sequences.

Recent advances in the field of algal genomics included only the complete sequencing of the nuclear genome of the microalgae *Cyanidioschyzon merolae*[53], *Ostreococcus tauri*[54], *Chlamydomonas reinhardtii*[55], and *Cyanophora paradoxa*[56] and the brown macroalga *Ectocarpus siliculosus*[57]. Moreover, EST projects have provided valuable information in the transcriptomic profile of some species of Rhodophyta [50,51,58-62] in the phylogenetic relationships among photosynthetic eukaryotes [63,64] and have also unveiled genes involved in stress response [52,65,66] and in life phase differentiation [67-70].

The transcriptomic profile of *L. dendroidea* and its corresponding associated microbiome was closely similar among all the samples, regardless of their place of origin. Likewise, a previous study verified a higher similarity between bacterial populations from seaweeds of the same species sampled at different sites than between those from different species growing at the same habitat, emphasizing the specificity of this association [71]. Our data reinforces these findings as we observed a high similarity in the taxonomic composition of the active microbiome associated with *L. dendroidea* in different sample sites.

### Major groups of transcripts of *L*. *dendroidea*

The functional annotation of the transcripts revealed predominantly basic cellular metabolic pathways. In general, functions related to translation and protein synthesis, from amino acid precursors to post-translational modifications are the most abundantly expressed in the transcriptome of *L*. *dendroidea*. Besides, complete pathways for energy production were well represented, mainly related to the pyruvate dehydrogenase complex, electron transfer, thioredoxins, citric acid cycle and NADH dehydrogenase. The ESTs involved in carbohydrate transport and metabolism (mainly glycolysis, starch and sucrose metabolism, and pentose phosphate pathway), Cofactors, Vitamins, Prosthetic Groups, Pigments (including Folate and Pterines, Tetrapyrroles and Pyridoxine), RNA Metabolism (mainly RNA Processing and Modification) were among the most represented categories in the transcriptome of *L*. *dendroidea*. Other relevant features in this transcriptome are related to DNA replication, recombination and repair, which are important to the survival and growth of the seaweed, especially in the rocky-shore coastal environment where the organisms are subject to high UVB levels that causes serious damages to DNA [72]. The ability to resist to UV-exposure influences the vertical distribution of seaweeds [73], and *L*. *dendroidea* typically grows in the lower midlittoral zone where UV-damage repair may be necessary. The same set of expressed sequences relevant in the transcriptome of *L*. *dendroidea* are among the most represented in the EST databases of *Gracilaria gracilis*[62], *G*. *changii*[51], *G*. *tenuistipitata*[60], *Porphyra yezoensis*[61,67], *P*. *haitanensis*[59], *Eucheuma denticulatum*[74], *Furcellaria lumbricalis*[52], and *Kappaphycus alvarezii*[66], possibly indicating a general pattern of expression in red seaweeds.

### Transcriptome of *L*. *dendroidea*-associated microbiome

Seaweeds are especially susceptible to epibiosis because they inhabit environments with strong competition for space [75], and release large amounts of organic compounds that induce the microbial colonization [76], but the interaction between seaweeds and their microbiomes is little known to the molecular level.

The functional analysis of the holobiont transcriptome revealed the expression of bacterial genes involved on cell motility and chemotaxis, for example the ESTs related to flagellum and CheY-like receiver domain which are important, respectively, for the recognition of the surface of the seaweed and the establishment of the biofilm [77,78]. However, the relatively low abundance of these transcripts in comparison with the ones involved in extracellular polysaccharide synthesis suggests a mature biofilm with some level of detachment, possibly of dispersal cells [79]. Transcripts coding for the enzyme S-adenosylmethionine synthetase, which participates in the synthesis of quorum sensing autoinducers, were also detected [80]. Quorum sensing (QS) is a bacterial cell to cell communication mechanism based on the release and perception of signaling molecules such as oligopeptides, N-acyl homoserine lactones (AHL) and autoinducers that allow bacteria to monitor their own population density and to coordinate swarming, biofilm formation, stress resistance, and biosynthesis of toxins and secondary metabolites [81], and it exhibits an important role in the interactions between bacteria and their eukaryotic hosts. Several red seaweeds are able to inhibit bacterial QS signaling, such as *Delisea pulchra*[82] and *Ahnfeltiopsis flabelliformis*[83], and a small inhibitory activity against QS signaling was previously detected in the ethyl acetate extract from a *Laurencia* sp. [84].

The taxonomic analysis of the transcriptome showed Bacteria as the dominant active group in the microbiome of *L*. *dendroidea*, with Cyanobacteria and Proteobacteria as the most represented bacterial phyla. These groups were also verified as predominant in the evaluation of the microbial diversity associated with four functional groups of seaweeds through metagenomics [17].

Among the cyanobacterial transcripts associated with the thalli of *L*. *dendroidea*, the Chroococcales, Oscillatoriales and Nostocales were the dominant orders, all of them comprising nitrogen fixing species. In a previous study, Phlips and Zeman [85] reported the occurrence and the nitrogen fixing activity of epiphytic forms of *Oscillatoria* associated to *Sargassum* thalli. Nitrogen can be the limiting nutrient in coastal ecosystems [86] and under this situation, nitrogen fixing cyanobacteria may be favored and gain in growth and reproductive success. In fact, Hoffman [87] pointed that despite their important contribution to benthic primary production, the main role of Cyanobacteria in the tropical marine ecosystems appears to be as nitrogen fixers. However, no sequences related to nitrogen fixation were observed in our data. This is expected since our data clearly indicates an oxygenic environment, and the nitrogenase expression is inhibited by oxygen [88]. Our samples, collected near the peak of photosynthetic activity (right before midday) should have a very low expression of this nitrogenase [89]. In fact, the most abundant cyanobacteria genus were *Synechococcus* and *Cyanothece*, which together with *Lyngbya* and *Synechocystis* were previously reported to rely on temporal separation between photosynthesis and nitrogen fixation, the last occurring mainly at night [90,91]. Further studies on the diel variation of the transcriptome profile could verify this hypothesis.

Analyzing the functional relative contribution of specific domains, we noticed a higher involvement of Bacteria in the Amino acid metabolism, except for the biosynthesis of glutamate, more represented in eukaryotes. Such situation was reported for *Rhizobium* nodules, where plants provide glutamate and a carbon source and in turn the nitrogen fixing Bacteria provide ammonium and amino acids such as alanine and aspartate for asparagine biosynthesis in the plant cytosol [92]. Although specialized mechanisms like nodules are not known in red algae, our data suggests a similar interaction between the seaweed and the associated microbiome, involving the exchange of nitrogen compounds.

Proteobacteria was the second largest active group with assigned sequences mostly to the classes Gammaproteobacteria and Alphaproteobacteria. The higher abundance of these classes was previously reported for the surface microbiome of the macroalgae *Ulva australis*[93] and *Laminaria hyperborean*[94], through denaturing gradient gel electrophoresis (DGGE) analysis. Predominantly heterotrophs, these groups would be opportunists, exploring an oxic productive environment [95]. The high prevalence of aerobic and aerotolerant groups reflects a photosynthesizing environment, also noted by Barott *et al.*[17]. The predominance of respiration over fermentative metabolism in the holobiont transcriptomic profile reinforces these findings. The aerobic metabolism generates reactive oxygen species (ROS) [96] that can damage DNA, lipids, and proteins [97]. In order to cope with oxygen toxicity and grow in aerobic conditions, Bacteria expressed genes correlated to oxidative stress, such as Superoxide dismutase, Glutaredoxins and Alkyl hydroperoxide reductase [98], and also stress related chaperones such as GroEL, DnaJ and DnaK [99,100].

Transcripts associated to photosynthesis and to the biosynthesis of carbohydrate reserves, such as starch, were more represented in eukaryotes, which indicate an important role of *L*. *dendroidea* in the primary production of the holobiont, generating carbon in excess to its immediate demand. The typical starch from Rhodophyta is called floridean starch and it shows structural similarities with starch granules from higher plants except for the lack of amylose in most of the species [101]. On the other hand the Bacteria contributed more to Carbohydrate and Lipid Transport and Metabolism, and to Energy Production and Conversion, standing out genes related to glycolysis and also to lipid and polysaccharide breakdown, reinforcing the role of Bacteria as consumers of organic matter in this holobiont [102].

Despite the beneficial or neutral interaction processes depicted here between *L*. *dendroidea* and its microbiome, some bacteria may also offer threats to the health and survival of seaweeds in their natural environment [103]. As such, defense mechanisms, such as the aforementioned secondary compounds of *L. dendroidea*[18], may have been evolutionarily selected. The expression of vanadium-dependent bromoperoxidases, involved on the halogenation and cyclization of terpenes in Rhodophyta [104], was detected in the transcriptomic profile of *L*. *dendroidea*. Additionally the previously reported increase on the bromination activity of red algae in response to infection signals, such as agar oligosaccharide [105], indicates an important role of this enzyme in the chemical defense of Rhodophyta.

### Terpenoid biosynthesis in the holobiont

The biosynthesis of terpenoid backbones provides precursors for the biosynthesis of diverse compounds that display relevant roles in plant and algal physiology [106]. The identified genes are involved in important steps for the biosynthesis of the building blocks dimethylallyl diphosphate, isopentenyl diphosphate and the higher-order building blocks geranyl diphosphate, farnesyl diphosphate and geranylgeranyl diphosphate, which are the precursors of monoterpenoids (C10), sesquiterpenoids (C15), and diterpenoids (C20), respectively [107]. The subsequent addition of isoprene units leads to the biosynthesis of sterols (isoprenoids with a C30 backbone) which are components of cell membranes; carotenoids (C40) and chlorophylls (with a C20 isoprenoid side-chain) that act as photosynthetic pigments; and plastoquinone, phylloquinone and ubiquinone (with long isoprenoid side-chains) that participate in electron transport systems for respiration or photosynthesis [106]. Terpenoid backbones are also required for the biosynthesis of N-glycans, important components for the proper folding of proteins in eukaryotic cells [108]. The biosynthesis of isopentenyl pyrophosphate (IPP) and dimethylallyl pyrophosphate (DMAPP), the central intermediates in the biosynthesis of isoprenoids, occur through two different pathways in plants, one dependent (MVA) and other independent of mevalonate (DOXP/MEP). The mevalonate (MVA) pathway, located in the cytosol, is responsible for the production of sterols, triterpenes and some sesquiterpenes [109]. The MVA-independent pathway operates in plastids and provides the precursors to monoterpenes, diterpenes, certain sesquiterpenes, carotenoids and the side chains of chlorophyll and plastoquinone [110]. This division between isoprenoids derived from plastids and cytoplasm was also observed in red algae [111,112]. Despite the occurrence of both biosynthetic routes in Rhodophyta, this study found only transcripts associated with the mevalonate-independent pathway. Furthermore, three transcripts were identified containing the terpene synthase family metal-binding domain [35], representing new possible targets for further functional clarification. Phylogenetic reconstruction based on genes of terpene synthases was attempted, using the fragments (50–310 amino acids) we obtained from our whole transcriptome strategy (data not shown). However, it is difficult to infer a phylogenetic relationship among taxonomic groups using the gene fragments of this pathway because, in nearly all cases, the bootstrap support for the branches is low when homologous sequences were available for analysis. Nevertheless, it is notable that in most cases, the sequences from *L*. *dendroidea* holobiont and other red algae cluster together with a relatively high bootstrap support.

These findings associated to the reconstruction of a complete pathway for the biosynthesis of terpenoid backbones in *L*. *dendroidea* are important steps to enable the heterologous biosynthesis of terpenes of interest, such as (-)-elatol, in genetically modified organisms. The molecular engineering of *Escherichia coli* and *Saccharomyces cerevisiae* has recently allowed the use of these microorganisms as cell factories to synthesize plant terpenes such as the antimalarial drug artemisinin [113,114], opening up new avenues for the scalable biosynthesis of terpenoid compounds. Our research provides a comparative basis for prospecting more specific terpene synthases genes for (-)-elatol and other commercially relevant terpenes, which could be explored in cell factories. This could be accomplished through the use of high producing strains of *L*. *dendroidea* under favorable conditions.

## Conclusions

Our work describes the first transcriptomic profile of the red seaweed *L*. *dendroidea*, increasing the knowledge of ESTs from the Florideophyceae class. Basic cellular metabolic functions were the most represented in this profile, as observed in other seaweeds. The associated microbial transcriptome was independent of the location of collect, and the holobiont transcriptome indicated interesting interactions such as biofilm formation, the possible exchange of nitrogen compounds between bacteria and eukaryotes, the role of *L*. *dendroidea* in photosynthesis and of bacteria as consumers of excess carbon, and the bacterial molecular strategies to cope with the oxidative stress generated during aerobic metabolism. In addition, seaweeds defense mechanisms were also suggested with the disclosure of a complete mevalonate-independent pathway. The present study is a first contribution to the transcriptomic analysis of *L*. *dendroidea*, and opens up new avenues for biotechnological applications using this seaweed.

## Competing interests

The authors declare that they have no competing interests.

## Authors’ contributions

LSO carried out the samples collection, and RNA extraction, participated in the bioinformatic analysis and drafted the manuscript. GBG participated in the bioinformatic analysis and in the discussions and draft of the manuscript. GGZS carried out the bioinformatic analysis and participated in the discussion of the results. LTS participated in the sample collection, the discussion of the results, and the acquisition of funding. GAF participated in the acquisition of funding, the work planning and the discussion of the results, MAF participated in RNA extraction, EST library construction and discussion of the results. RCP participated in the acquisition of funding, work planning, discussion of the results, and draft of the manuscript. FLT participated in the acquisition of funding, work planning, data interpretation and draft of the manuscript. All authors read and approved the final manuscript.

## Supplementary Material

Additional file 1 COG functional profile of the transcriptome of *L. dendroidea* (separate samples). Click here for file

Additional file 2 Bacterial phyla recognized on the transcriptome of *L. dendroidea* (separate samples). Click here for file

Additional file 3 Relevant functions for the interaction between Bacteria and Eukarya in the transcriptomic profile of the holobiont. Click here for file
