# Supplementary figures and images for: Transcriptomic analysis of the red seaweed Laurencia dendroidea (Florideophyceae, Rhodophyta) and its microbiome
Source: BMC Genomics. 2012 Sep 17;13:487. doi: 10.1186/1471-2164-13-487 (PMC3534612; doi:10.1186/1471-2164-13-487)

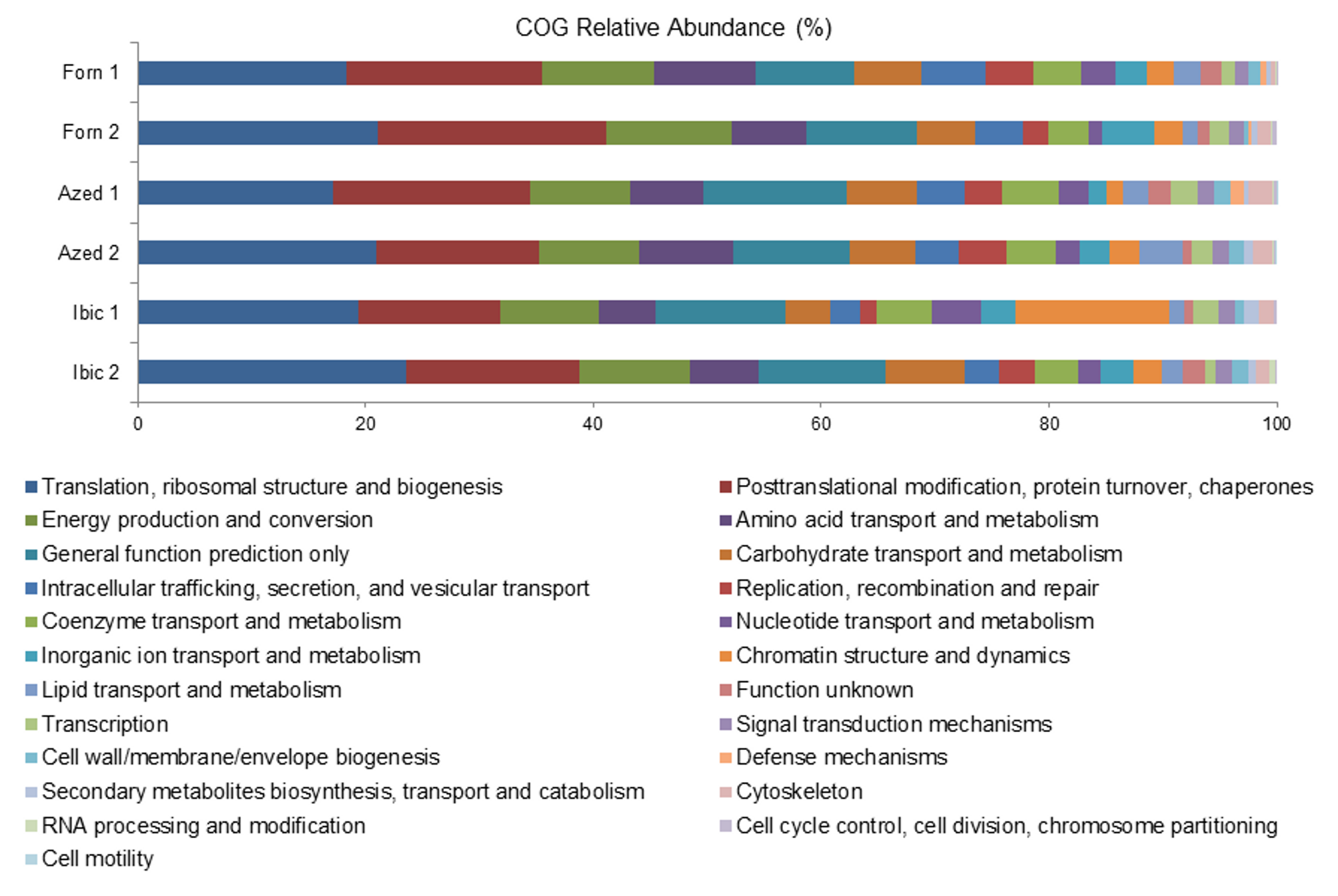

Supplement: Additional file 1 — COG functional profile of the transcriptome of L. dendroidea (separate samples). [file 1471-2164-13-487-S1.tiff]

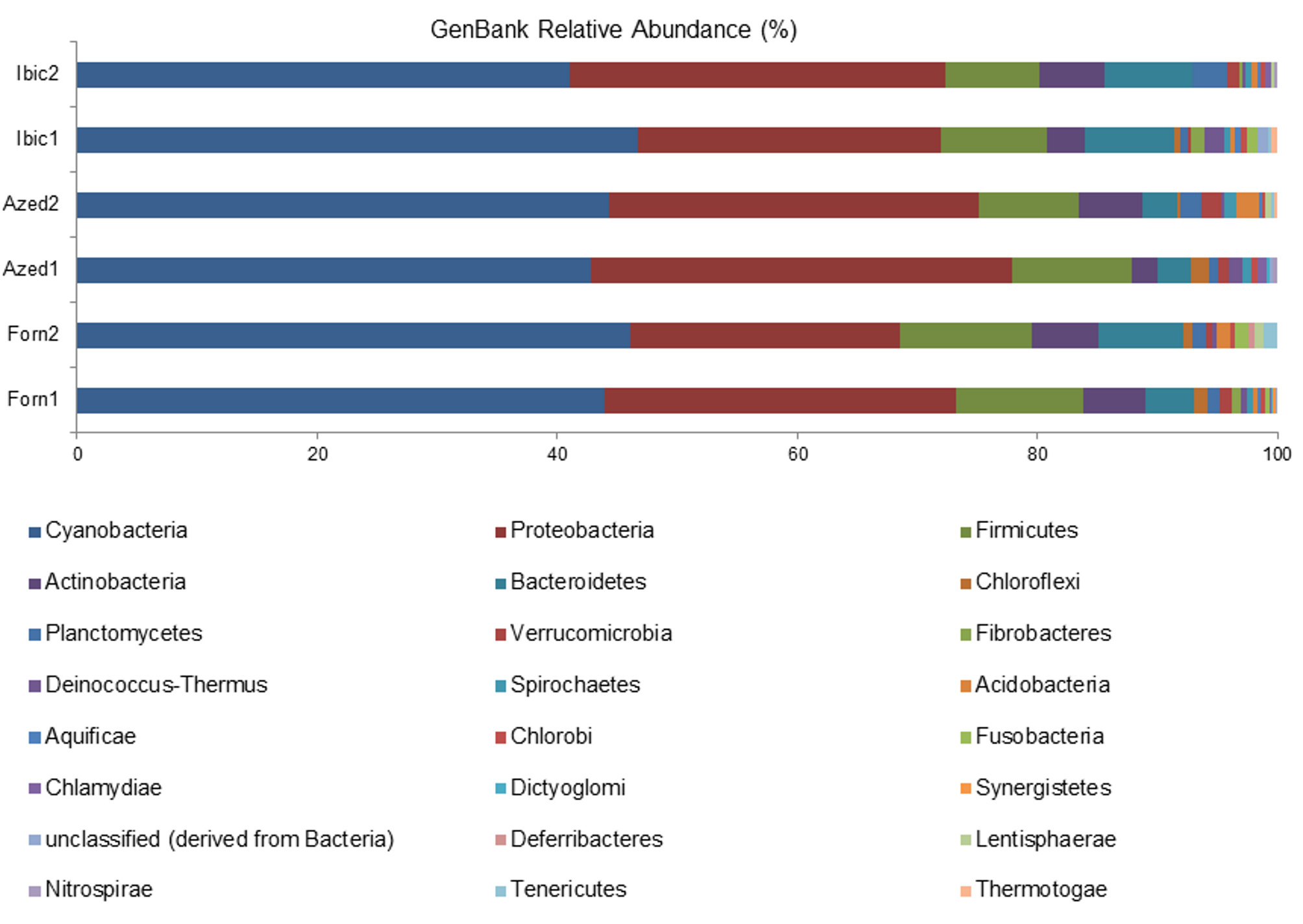

Supplement: Additional file 2 — Bacterial phyla recognized on the transcriptome of L. dendroidea (separate samples). [file 1471-2164-13-487-S2.tiff]
