# Supplementary material for: Transcriptomic analysis of the red seaweed Laurencia dendroidea (Florideophyceae, Rhodophyta) and its microbiome
Source: BMC Genomics. 2012 Sep 17;13:487. doi: 10.1186/1471-2164-13-487 (PMC3534612; doi:10.1186/1471-2164-13-487)
Supplement: Additional file 3 — Relevant functions for the interaction between Bacteria and Eukarya in the transcriptomic profile of the holobiont. [file 1471-2164-13-487-S3.docx]

| Function | Eukarya | Bacteria | p-value |
| --- | --- | --- | --- |
| Heat shock protein 60 family chaperone GroEL | **0** | **3.171** | **4.89E-06** |
| Chaperone protein DnaJ | **0** | **1.057** | **0.019** |
| Chaperone protein DnaK | 0 | 0.846 | 0.057 |
| Manganese superoxide dismutase | 0 | 0.528 | 0.172 |
| Glutaredoxins | 0 | 0.423 | 0.305 |
| Alkyl hydroperoxide reductase | 0 | 0.210 | 0.548 |
| Photosynthesis | **3.179** | **1.578** | **0.011** |
| starch and sucrose metabolism (synthesis) | **0.663** | **0** | **0.0257** |
| Carbohydrate transport and metabolism | **3.480** | **5.626** | **0.0307** |
| Lipid transport and metabolism | **1.408** | **3.580** | **0.002** |
| Energy production and conversion | **5.799** | **11.381** | **1.42E-05** |
| Amino acid transport and metabolism | **3.645** | **11.509** | **2.84E-11** |
| Glutamate synthase domain 2 | **0.580** | **0** | **0.047** |

Bold values indicate statistical difference between domains.
